# Effects of *Bifidobacterium*-Fermented Milk on Obesity: Improved Lipid Metabolism through Suppression of Lipogenesis and Enhanced Muscle Metabolism

**DOI:** 10.3390/ijms25189934

**Published:** 2024-09-14

**Authors:** Hitomi Maruta, Yusuke Fujii, Naoki Toyokawa, Shoji Nakamura, Hiromi Yamashita

**Affiliations:** 1 Department of Nutritional Science, Faculty of Health and Welfare Science, Okayama Prefectural University, 111 Kuboki, Soja-shi 719-1197, Okayama, Japan; maruta@fhw.oka-pu.ac.jp; 2 Fundamental Laboratory, Ohayo Daily Products Co., Ltd., 565 Koshita, Naka-ku, Okayama-shi 703-8505, Okayama, Japan; y_fujii@ohayo-milk.com (Y.F.); naoki.toyokawa@rc-iplaw.com (N.T.); nakanakasyo1121@ymobile.ne.jp (S.N.); 3 Graduate School of Health and Welfare Science, Okayama Prefectural University, 111 Kuboki, Soja-shi 719-1197, Okayama, Japan

**Keywords:** *Bifidobacterium*, acetic acid, AMPK, anti-obesity, lipid metabolism, skeletal muscles, mitochondria

## Abstract

Obesity is a major global health concern. Studies suggest that the gut microflora may play a role in protecting against obesity. Probiotics, including lactic acid bacteria and *Bifidobacterium*, have garnered attention for their potential in obesity prevention. However, the effects of *Bifidobacterium*-fermented products on obesity have not been thoroughly elucidated. *Bifidobacterium*, which exists in the gut of animals, is known to enhance lipid metabolism. During fermentation, it produces acetic acid, which has been reported to improve glucose tolerance and insulin resistance, and exhibit anti-obesity and anti-diabetic effects. Functional foods have been very popular around the world, and fermented milk is a good candidate for enrichment with probiotics. In this study, we aim to evaluate the beneficial effects of milks fermented with *Bifidobacterium* strains on energy metabolism and obesity prevention. Three *Bifidobacterium* strains (Bif-15, Bif-30, and Bif-39), isolated from newborn human feces, were assessed for their acetic acid production and viability in milk. These strains were used to ferment milk. Otsuka–Long–Evans Tokushima Fatty (OLETF) rats administered Bif-15-fermented milk showed significantly lower weight gain compared to those in the water group. The phosphorylation of AMPK was increased and the expression of lipogenic genes was suppressed in the liver of rats given Bif-15-fermented milk. Additionally, gene expression related to respiratory metabolism was significantly increased in the soleus muscle of rats given Bif-15-fermented milk. These findings suggest that milk fermented with the *Bifidobacterium* strain Bif-15 can improve lipid metabolism and suppress obesity.

## 1. Introduction

The prevalence of obesity has been increasing worldwide. World Health Organization reported that, as of 2022, one in eight people is affected by obesity. Obesity is linked to several health conditions, including type 2 diabetes, dyslipidemia, and hypertension. Recent studies have suggested a connection between the gut microbiota and obesity-related disorders, such as type 2 diabetes. Probiotics have attracted attention for an important role in maintaining good health [1,2,3], such as probiotic strains of *Bifidobacterium longum*, which enhances gastrointestinal health [4]; *Lactobacillus casei* strain Shirota, which improves insulin resistance and glucose intolerance [5]; and *Lactobacillus* strains, which are effective in cholesterol removal [6]. Probiotics, particularly *Lactobacillus* and *Bifidobacterium*, are essential for understanding the gut microbiota balance and managing obesity [7,8,9]. The intestinal microbiota produces short-chain fatty acids (SCFAs), including acetic acid, propionic acid, and butyric acid, through the fermentation of starches, unabsorbed sugars, cellulosic and non-cellulosic polysaccharides, and mucins [8].

*Bifidobacterium*, found in the guts of various animals, is a well-known probiotic bacteria, and its beneficial effects on human health have been reported. The *Bifidobacterium longum* BB536 (*B. longum* BB536) strain has been reported to have various physiological benefits, including anti-allergic effects [10,11,12]; reduction of harmful bacteria [13,14]; improvements in intestinal health, defecation frequency, and stool characteristics [15,16]; and reductions in total cholesterol levels, liver lipid deposition, and adipocyte size [17,18]. Moreover, *Bifidobacterium longum* shows an anti-obesity effect on high-fat-diet-induced obese rats [19]. The preventive effect of probiotic strains of *Bifidobacterium longum* on enterohemorrhagic *Escherichia coli* infection is attributed to the high carbohydrate metabolism of these strains, which leads to acetate production and upregulates the barrier function of the host gut epithelium [20].

*Bifidobacterium* produces acetic acid as a final metabolite [21,22], which has been shown to improve glucose tolerance and insulin resistance and has anti-obesity and anti-diabetic properties [23,24]. Administering acetic acid to Otsuka–Long–Evans Tokushima Fatty (OLETF) rats, an obese and type 2 diabetic model, reduces body weight gain and suppresses the transcription of lipogenic genes such as ACC, ME, G6PD, L-PK, and FAS [23]. Acetic acid supplementation also stimulates lipid metabolism in skeletal muscles and reduces lipid accumulation in the adipose tissue of OLETF rats [23,25].

*Bifidobacterium* is used globally as a probiotic in various food products, including yogurt, milk, or dietary supplements [26,27]. The probiotic products contain high enough viable counts at the end of their shelf-life. Among the many foods available containing *Bifidobacterium*, yogurt or fermented milk is easy to take regularly and it may be the most common product [16]. As of 1986, conventional yogurt cultures have been replaced by *Bifidobacterium* species to enhance the health benefits of yogurt [17]. Supplementation of *Bifidobacterium*-fermented milk has effects on improving the intestinal environment, fecal characteristics and defecation frequency [16]; on decreasing total cholesterol, LDL-cholesterol, and triglyceride levels; and on increasing the HDL-cholesterol concentration [18]. 

In this study, we investigated milks fermented with novel *Bifidobacterium* strains, which were isolated from the feces of newborn humans, for their effects on lipid metabolism and obesity prevention in OLETF rats.

## 2. Results

### 2.1. Milk Fermented with the Bifidobacterium Bif-15 Strain Prevents Obesity 

**Figure 1:**
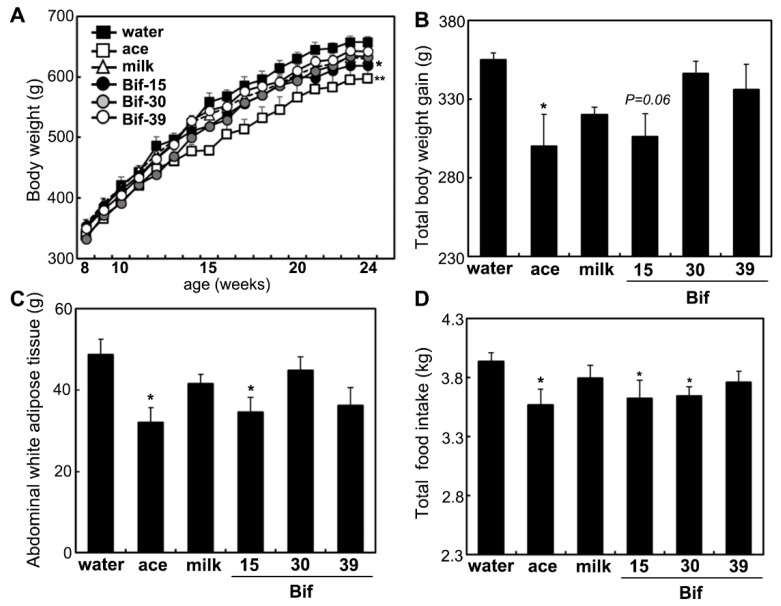
Total body weight gain and total food intake. (**A**) Changes in body weight during the intervention period, (**B**) total body weight gain, (**C**) abdominal white adipose tissue weight, and (**D**) total food intake. Body weight changes in rats administered distilled water (water), acetic acid (ace), skim milk (milk), or *Bifidobacterium*-fermented milk (Bif-15, Bif-30, and Bif-39) starting from 15 weeks of age. Each value represents the mean ± SE (*n* = 4). * *p* < 0.05 and ** *p* < 0.01, according to Dunnett’s test, compared to the water group.

Body weight at 24 weeks of age was significantly lower in the acetic acid and Bif-15 groups of rats compared to the water group (Figure 1A). Body weight gain was significantly reduced in the acetic acid group and tended to be lower in the Bif-15 group compared to the water group (Figure 1B). Abdominal white adipose tissue weight was significantly decreased in both the acetic acid and Bif-15 groups (Figure 1C). Total food intake was significantly lower in rats from the acetic acid, Bif-15, and Bif-30 groups compared to the water group (Figure 1D).

### 2.2. Long-Term Supplementation with Bif-15-Fermented Milk Increases Plasma Acetic Acid and HDL-C Levels

Plasma acetic acid and HDL-cholesterol levels were significantly increased in the Bif-15 group compared to the control group (Table 1). 

### 2.3. Effects of Acetic Acid and Bifidobacterium-Fermented Milk on AMPK Phosphorylation in the Liver

**Figure 2:**
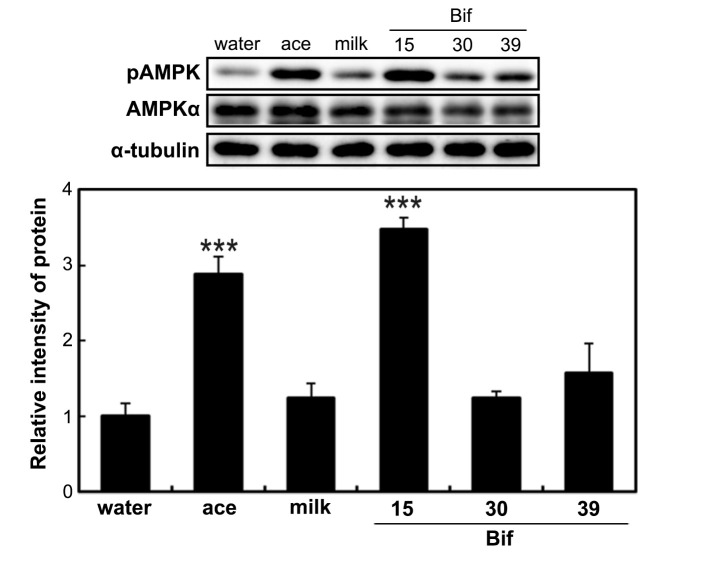
Effect of *Bifidobacterium*-fermented milks on the phosphorylation of AMPK in the liver. Total protein was isolated from the livers of OLETF rats in the water, ace, milk, Bif-15, Bif-30, and Bif-39 groups, as described in the Section 4. Western blotting was performed to determine AMP-activated protein kinase (AMPK) phosphorylation in the liver. Each value represents the mean ± SE (*n* = 4). *** *p* < 0.001, according to Dunnett’s test, compared to the water group.

To analyze AMPK phosphorylation following supplementation with *Bifidobacterium*-fermented milk, the phosphorylated AMPK level in the liver was assessed. AMPK phosphorylation was increased in the livers of rats from the acetic acid and Bif-15 groups (Figure 2).

### 2.4. Bif-15-Fermented Milk Suppresses the Expression of Lipogenic Genes in the Liver

**Figure 3:**
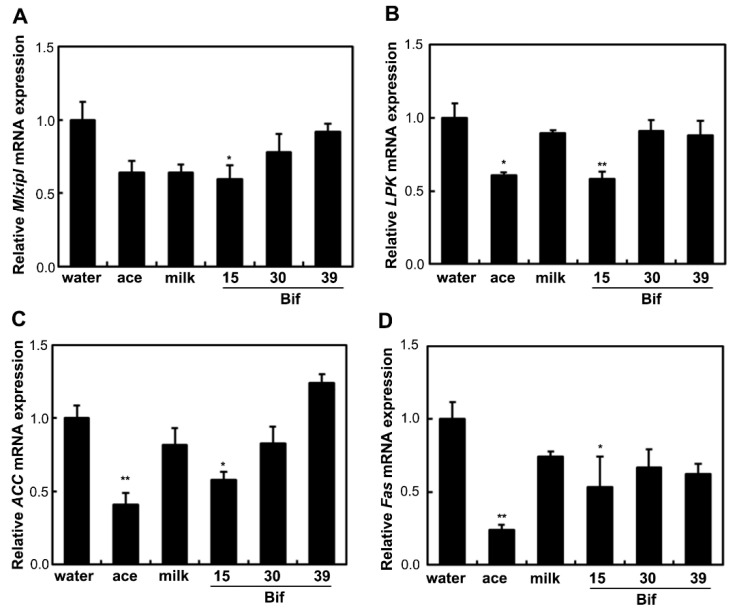
Effects of *Bifidobacterium*-fermented milks on mRNA levels in the liver. Total RNA was isolated from the liver at 24 weeks of age. Real-time PCR analysis was performed to determine the mRNA levels of *Mixipl* (**A**), *LPK* (**B**), *ACC* (**C**), and *Fas* (**D**) in the liver of rats from the water, ace, milk, Bif-15, Bif-30, and Bif-39 groups. Each value represents the mean ± SE (*n* = 4). * *p* < 0.05 and ** *p* < 0.01, according to Dunnett’s test, compared to the water group.

To examine the effects of *Bifidobacterium*-fermented milk on lipogenic gene expression in the liver, the expression levels of the ChREBP (*Mlxipl*), LPK (*LPK*), ACC (*ACC*), and FAS (*Fas*) genes were analyzed. Supplementation with acetic acid and Bif-15-fermented milk reduced the expression of *LPK*, *ACC*, and *Fas* compared to the water group (Figure 3). Bif-15-fermented milk also suppressed the expression of the ChREBP gene compared to the water group. 

### 2.5. Effect of Bifidobacterium Bif-15-Fermented Milk on Fatty Liver Modulation

**Figure 4:**
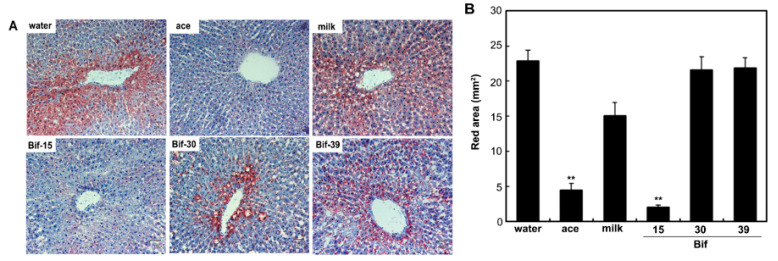
Histological sections of the liver. (**A**) Representative images of Oil Red O staining (×100 magnification, scale bar = 500 μm) in the livers of OLETF rats from the water, ace, milk, Bif-15, Bif-30, and Bif-39 groups. (**B**) Red areas (lipid droplets) were measured using ImageJ software. ** *p* < 0.01, according to Dunnett’s test, compared to the water group.

To determine if *Bifidobacterium*-fermented milk modulates lipid accumulation in the liver, a histological analysis was performed. Supplementation with acetic acid and Bif-15-fermented milk protected against lipid accumulation in the liver compared to the water group (Figure 4).

### 2.6. Effects of Bif-15 Supplementation on Gene and Protein Expression in Skeletal Muscles 

**Figure 5:**
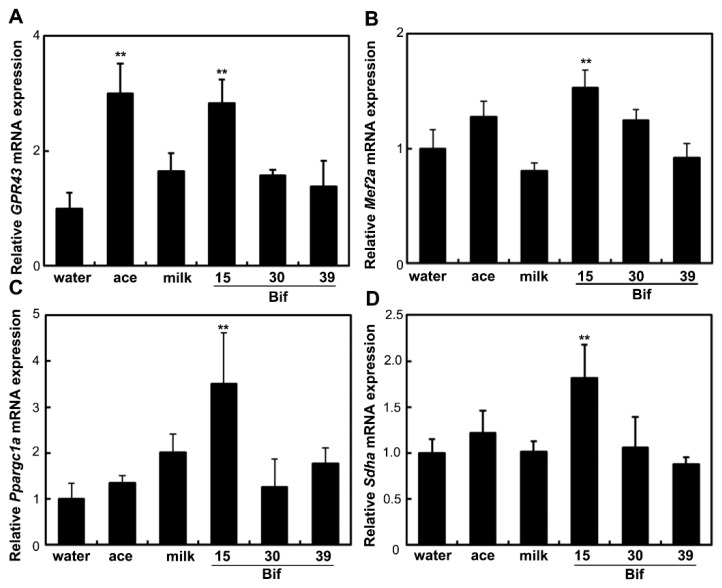
Effects of *Bifidobacterium*-fermented milks on the expression of the GPR43 (**A**), MEF2A (**B**), PGC-1α (**C**), and SDH (**D**) genes in the soleus muscle. Total RNA was isolated from the soleus muscle of OLETF rats in the water, ace, milk, Bif-15, Bif-30, and Bif-39 groups at 24 weeks of age. Real-time PCR analysis was performed to determine the mRNA levels of *GPR43*, *Mef2a*, *Ppargc1a*, and *Sdha* in the soleus muscle. Each value represents the mean ± SE (*n* = 4). ** *p* < 0.01, according to Dunnett’s test, compared to the water group.

**Figure 6:**
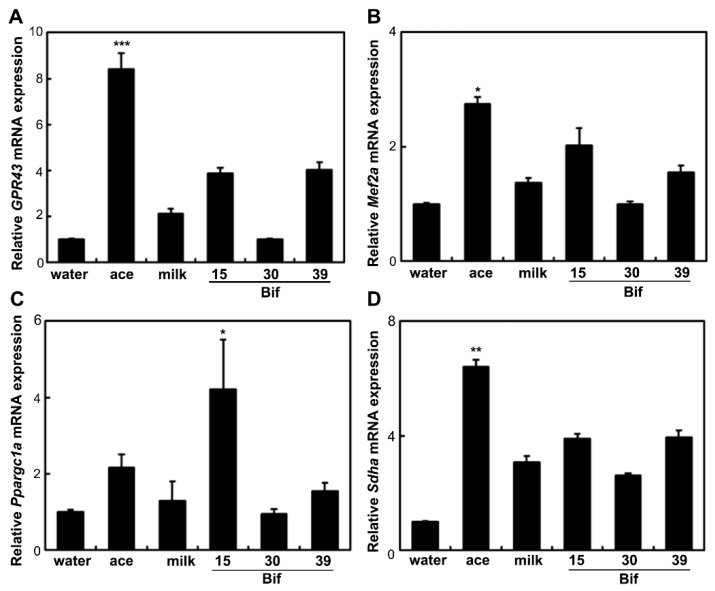
Effects of *Bifibacterium* fermented milks on the expression of the GPR43 (**A**), MEF2A (**B**), PGC-1α (**C**), and SDH (**D**) genes in the gastrocnemius muscle of rats. Total RNA was isolated from the gastrocnemius muscle of OLETF rats at 24 weeks of age. Real-time PCR analysis was performed to determine the mRNA levels of *GPR43*, *Mef2a*, *Ppargc1a*, and *Sdha* in the gastrocnemius muscle. Each value represents the mean ± SE (*n* = 4). * *p* < 0.05, ** *p* < 0.01, and *** *p* < 0.001, according to Dunnett’s test, compared to the water group.

**Figure 7:**
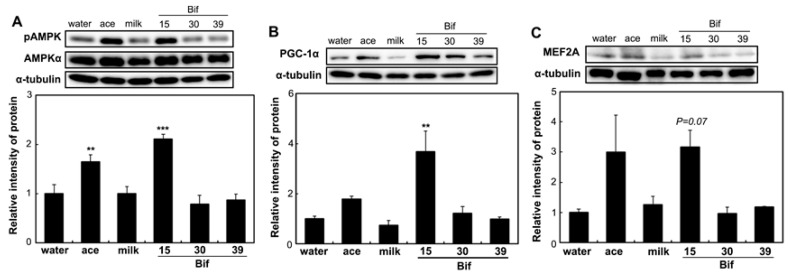
Effects of *Bifibacterium*-fermented milks on the phosphorylated AMPK, PGC-1α, and MEF2A protein levels in the soleus muscle of rats. Total protein was isolated from the soleus muscle of OLETF rats in the water, ace, milk, Bif-15, Bif-30, and Bif-39 groups at 24 weeks of age. Western blotting was carried out to determine the levels of pAMPK (**A**), PGC-1α (**B**), and MEF2A (**C**), as described in the Section 4. Each value represents the mean ± SE (*n* = 4). ** *p* < 0.01, and *** *p* < 0.001, according to Dunnett’s test, compared to the water group.

**Figure 8:**
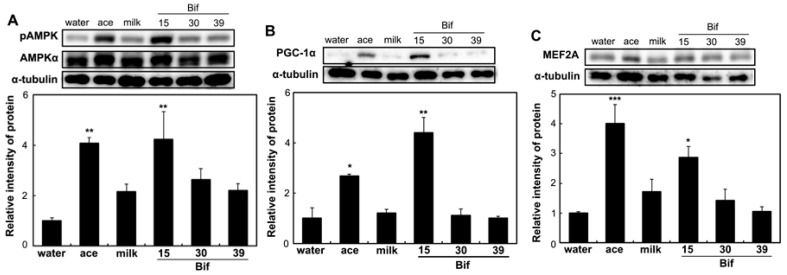
Effects of *Bifibacterium*-fermented milks on the phosphorylated AMPK, PGC-1α, and MEF2A protein levels in the gastrocnemius muscle of rats. Total protein was isolated from the gastrocnemius muscles of OLETF rats in the water, ace, milk, Bif-15, Bif-30, and Bif-39 groups at 24 weeks of age. Western blotting was carried out to determine the levels of pAMPK (**A**), PGC-1α (**B**), and MEF2A (**C**), as described in the Section 4. Each value represents the mean ± SE (*n* = 4). * *p* < 0.05, ** *p* < 0.01, and *** *p* < 0.001, according to Dunnett’s test, compared to the water group.

**Figure 9:**
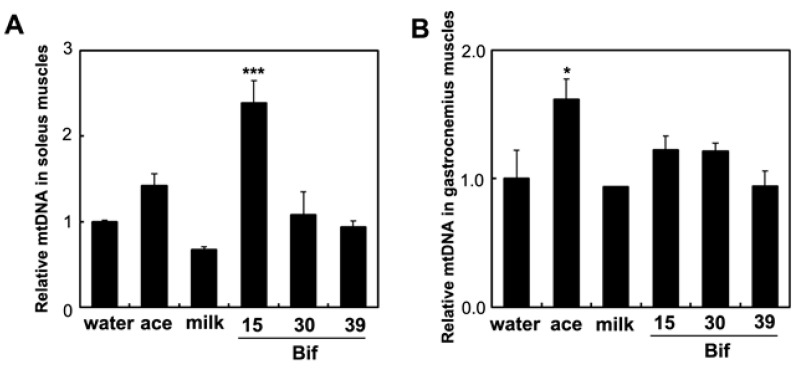
Effects of *Bifibacterium*-fermented milks on the mtDNA levels in the skeletal muscles. Genomic DNA was isolated from the soleus and gastrocnemius muscles of OLETF rats in the water, ace, milk, Bif-15, Bif-30, and Bif-39 groups at 24 weeks of age. Real-time PCR analysis was performed to determine *ND1* levels in the soleus (**A**) and gastrocnemius (**B**) muscles. Each value represents the mean ± SE (*n* = 4). * *p* < 0.05 and *** *p* < 0.001, according to Dunnett’s test, compared to the water group.

To assess the impact of Bif-15-fermented milk on skeletal muscle function, the expression of genes associated with energy metabolism in skeletal muscles was analyzed. In the soleus muscle, supplementation with Bif-15-fermented milk increased the expression of the GPR43 (*ffar2*), MEF2A (*mef2a*), PGC-1α (*ppargc1a*), and SDH (*sdha*) genes (Figure 5). Acetic acid supplementation increased the expression of *GPR43*. In the gastrocnemius muscle, Bif-15-fermented milk increased the expression of *ppargc1a* (Figure 6C), while acetic acid supplementation increased the expression of *GPR43*, *mef2a*, and *sdha* (Figure 6A,B,D). Both acetic acid and Bif-15-fermented milk supplementation increased AMPK phosphorylation in the soleus muscle (Figure 7A). PGC-1α protein levels in the soleus muscle were elevated in the Bif-15 group (Figure 7B). In the gastrocnemius muscle, the phosphorylation of the AMPK, PGC-1α, and MEF2A proteins increased in the acetic acid and Bif-15 groups (Figure 8). The mitochondrial DNA (mtDNA) level in the soleus muscle was also higher in the Bif-15 group compared to the water group, and the mtDNA level in the gastrocnemius muscle increased in the acetic acid group (Figure 9A,B).

## 3. Discussion

*Bifidobacterium* exist in the gut of animals and is known to improve lipid metabolism [17,19,28]. In this study, we investigated the effects of supplementing three types of *Bifidobacterium* strains in fermented milk on lipid metabolism and obesity protection in OLETF rats.

Rats supplemented with Bif-15 strain-fermented milk gained less body weight, similar to the acetic acid group, compared to the water group (Figure 1A). The plasma acetic acid concentration was higher in the Bif-15 group than in the water group (Table 1). Although all three *Bifidobacterium* strains produced approximately 0.5% acetic acid during milk fermentation (Table 2), Bif-15 generated acetic acid significantly in the gut, which then entered the bloodstream after supplementation (Table 1). Liver lipid accumulation was reduced in the Bif-15 group, similar to the acetic acid group, compared to the water group (Figure 4A,B). The phosphorylated AMPK level was significantly higher in the Bif-15 group, similar to the acetic acid group, compared to the water group (Figure 2). The expression of the ChREBP gene, which regulates lipogenesis from glucose in the liver, was significantly lower in the Bif-15 group compared to the water group (Figure 3A). Additionally, the expression levels of the LPK, ACC, and FAS genes decreased in the livers of Bif-15-supplemented rats, similar to the effect observed in the acetic acid group (Figure 3B–D). These results suggest that fermented milk supplemented with Bif-15 can suppress liver lipid generation and enhance lipid metabolism. Moreover, the expression of the GPR43, MEF2A, PGC-1α, and SDH genes, which are associated with respiratory metabolism, was significantly increased in the soleus muscles of Bif-15-supplemented rats compared to the water group (Figure 5). MEF2A is a transcription factor involved in skeletal muscle differentiation and is associated with the stimulation of type I fiber proteins [29]. PGC-1α plays a crucial role in regulating mitochondrial biogenesis and oxidative metabolism, driving the formation of slow-twitch fibers [30]. SDH is a mitochondrial marker enzyme [31,32,33]. GPR43 is a G-protein-coupled receptor linked to energy metabolism and mitochondrial function [34,35,36]. Acetic acid acts as a signaling molecule that activates GPR43 [37,38,39,40]. Our previous study showed that acetic acid treatment induced the expression of several genes associated with slow-twitch fibers in L6 myotube cells, including those encoding MEF2A, myoglobin, PGC-1α, and SDH, through the activation of AMPK and GPR43 [41,42]. Acetic acid activates GPR43 and induces calcium influx, leading to the proliferation of slow-twitch fibers in L6 cells [42]. Furthermore, GPR43 gene expression is stimulated by acetic acid treatment in the soleus muscles of aging rats [43]. In this study, GPR43 expression was significantly higher in the soleus muscle of rats treated with Bif-15 and acetic acid compared to the water group (Figure 5A). The phosphorylation of AMPK was also significantly increased in the soleus muscle of rats in the Bif-15 group, similar to the effect observed on the acetic acid group, compared to the water group (Figure 7A). The mitochondrial DNA level was increased in the soleus muscle of rats in the Bif-15 group compared to the water group (Figure 9A). These results suggest that Bif-15-fermented milk might have more effects on improving mitochondrial function and respiratory metabolism in the soleus muscle as compared with acetic acid. In previous reports, heat-killed *Bifidobacterium* had effects on the muscle mass and mitochondrial biogenesis [44,45], indicating that the bacterial cells might have effects on the regulation of metabolic genes, other than the effect of generating acetic acid. In the gastrocnemius muscle, the expression of the GPR43, Mef2A, and SDH genes was significantly higher in the acetic acid group, while the expression of the PGC-1α gene was significantly increased in the Bif-15 group (Figure 6A–D). In the gastrocnemius muscle, the phosphorylated AMPK level and the expression of PGC-1α and MEF2A proteins were significantly higher in both the Bif-15 and acetic acid groups compared to the water group (Figure 8). The mitochondrial DNA level in the gastrocnemius muscle was higher in the acetic acid group compared to the water group (Figure 9B). Previous studies have shown that acetic acid treatment modulates mitochondrial function [42,43]. In the gastrocnemius muscle, the effect of Bif-15-fermented milk on the expression of genes associated with respiratory metabolism was not similar to that of acetic acid, while the effect on the expression of proteins was similar in the two groups compared to the water group. The mechanism of the effect of Bif-15-fermented milk is not clear at this point and further study is needed to clearly identify the contributing factors for the regulation of metabolic genes and proteins in the gastrocnemius muscle. In conclusion, these findings suggest that milk fermented with the Bif-15 strain may produce acetic acid in the gut, contributing to improved lipid and respiratory metabolism in the liver and skeletal muscles of OLETF rats. Other strains, such as Bif-30 and Bif-39, may be less effective at generating acetic acid in the gut compared to Bif-15. The mechanism by which Bif-15 produces acetic acid in the gut remains unclear. Blood acetic acid levels might influence liver and skeletal muscle functions. However, other factors, such as *Bifidobacterium* cells, might have effects on the regulation of energy metabolism. Further research is needed to elucidate the mechanisms underlying the effects of Bif-15-fermented milk on the prevention of obesity and to determine its potential for clinical trials. 

## 4. Materials and Methods

### 4.1. Materials

Isopentane, formaldehyde, 2-mercaptoethanol, and a 1% eosin Y solution were purchased from FUJIFILM Wako Pure Chemical Corporation (Osaka, Japan). 

### 4.2. Preparation of Bifidobacterium Strains

Three *Bifidobacterium* strains (Bif-15, Bif-30, and Bif-39) were isolated from human infant feces. The *Bifidobacterium* strains used in the experiments were identified as *Bifidobacterium longum* (Bif-15), *Bifidobacterium* sp. (Bif-30), and *Bifidobacterium pseudocatenulatum* (Bif-39) by phenotypic testing and an analysis of the upstream 500 bp of the 16S rRNA gene. These strains were confirmed to have high acetic acid production and good viability in milk. Each strain was cultured in GAM broth and then inoculated into 12% reconstituted skim milk, where it was cultured until the acetic acid concentration reached approximately 0.5%. The fermented milk was used for animal experiments. The characterization of these *Bifidobacterium* strains and the composition of organic acids in the fermented milk are detailed in Table 2.

### 4.3. Animal Experiments

All animal experiments were conducted per the guidelines of Okayama Prefectural University and the relevant Japanese laws and notifications. Approval was obtained from the Animal Care and Use Committee of Okayama Prefectural University (protocol number 29-2). Five-week-old male OLETF rats, a genetic model exhibiting lipid accumulation, obesity, and spontaneous development of non-insulin-dependent diabetes mellitus (Hoshino Laboratory Animals, Ibaraki, Japan), were used. The rats were fed a normal laboratory diet (CE-2; CLEA Japan, Inc., Tokyo, Japan) for two weeks to stabilize their metabolic conditions. They were housed individually in an air-conditioned room at approximately 25 °C with a 12 h light/dark cycle (light from 08:00 to 20:00). All animals had free access to water and food. The rats were randomly assigned to one of the following treatment groups: water, acetic acid, skim milk (milk), or *Bifidobacterium* strain Bif-15-, Bif-30-, or Bif-39-fermented milk. The water group received distilled water orally, the acetic acid group received 1% (*v/v*) acetic acid, and the milk group received 12% reconstituted skim milk (Yotsuba Milk Products Co., Ltd., Hokkaido, Japan). Rats in the Bif-15, Bif-30, and Bif-39 groups were administered 5 mL/kg body weight (BW) of milk fermented with *Bifidobacterium* strains Bif-15, Bif-30, and Bif-39, respectively, daily for 5 days a week until 24 weeks of age. Food consumption and BW were recorded daily. At 24 weeks of age, rats were anesthetized with an intraperitoneal injection of pentobarbital (Sumitomo Dainippon Pharma, Tokyo, Japan). Tissue samples were collected 24 h after the final administration in the fed state. White adipose tissue (WAT), liver, gastrocnemius muscle, and soleus muscle were immediately isolated, weighed, frozen in liquid nitrogen, and stored at −80 °C for subsequent analyses.

### 4.4. Blood Biochemical Analysis

Blood samples were collected from the inferior vena cava. Plasma was separated by centrifuging the samples at 3000× *g* for 15 min at 4 °C (MX-305, TOMY, Tokyo, Japan). Plasma glucose, triglyceride (TG), total cholesterol (TC), and high-density lipoprotein-cholesterol (HDL-C) levels were measured using an enzymatic method (Wako Assay Kit; FUJIFILM Wako, Osaka, Japan). Plasma acetic acid levels were measured using an enzymatic method [46].

### 4.5. Histological Analysis

Small liver tissue pieces were quickly frozen in O.C.P. compound (Sakura, Kyoto, Japan). Cryostat sections (10 μm thick) were obtained and stained with hematoxylin and eosin and Oil Red O. Images were captured with a CCD camera (Olympus, Tokyo, Japan) at a magnification of ×100. Red areas (lipid droplets) were measured using ImageJ software.

### 4.6. Quantitative RT-PCR Analysis

Total RNA and genomic DNA were extracted from isolated skeletal muscles using Sepasol-RNA Super I (Nacalai Tesque, Kyoto, Japan) and extraction buffer (4 M guanidine thiocyanate, 50 mM sodium citrate, and 1 M Tris). Total RNA was reverse transcribed using the ReverTra Ace qPCR Master Mix and a gDNA remover kit (TOYOBO, Osaka, Japan), according to the manufacturer’s instructions. Real-time quantitative PCR analyses were performed using a StepOnePlus detection system (Thermo Fisher Scientific, Applied Biosystems, CA, USA) with KAPA SYBR FAST qPCR Kits (Kapa Biosystems, Wilmington, MA). Primer sequences used for amplification are listed in Table 3.

### 4.7. Western Blotting

Rat tissues were homogenized in extraction buffer (2.5 mM Tris [pH 8.0], 0.5 mM EDTA, 10 mM MgCl₂, and 0.25 M sucrose). The homogenate was centrifuged (2150× *g*, 10 min, 4 °C) to remove tissue debris. The protein content in the supernatant was determined by the Bradford assay, and 30 μg of protein from each tissue extract was used for Western blot analysis to determine the levels of AMPKα phosphorylated at Thr-172 (Cell Signaling, MA, USA), AMPKα (Cell Signaling, MA, USA), MEF2A (Santa Cruz, TX, USA), PGC-1α (Santa Cruz, TX, USA), and α-tubulin (FUJIFILM Wako, Osaka, Japan). Samples were subjected to 10% SDS-PAGE, and proteins were transferred onto a polyvinylidene difluoride membrane (Merck KGaA, DA, Germany). Membranes were incubated with primary antibodies overnight at 4 °C, washed three times with TBST (2.5 mM Tris-HCl, 13.8 mM NaCl, 0.27 mM KCl, and 0.05% Tween20 [pH 7.6]), and then incubated with HRP-conjugated secondary antibodies, goat anti-mouse IgG H&L (ab6789) and goat anti-rabbit IgG H&L (ab6721) (Abcam plc., Cambridge, UK), for 60 min. After washing three times with TBST, chemiluminescence was detected using ImmunoStar LD (FUJIFILM Wako, Osaka, Japan) following the manufacturer’s protocol. Signals were visualized and quantified using ImageQuant LAS-4000 and Multi Gauge V3.2 analysis software (Fujifilm, Tokyo, Japan).

### 4.8. Statistical Analysis

All statistical analyses were performed using one-way ANOVA followed by Dunnett’s multiple comparisons test (* *p* < 0.05, ** *p* < 0.01, and *** *p* < 0.001 relative to the water control) with IBM SPSS Statistics for Windows, version 27.0 (IBM Corp., Armonk, NY, USA). 

## Figures and Tables

**Table 1:** Biochemical assay results for plasma.

	Glucose (mg/dL)	TG (mg/dL)	TC (mig/dL)	HDL-C (mg/dL)	Insulin (ng/mL)	Acetic Acid (μM)
Water	258 ± 33	350 ± 10	210 ± 3.1	69 ± 5.3	7.0 ± 2.2	94 ± 11
Ace	263 ± 6.5	210 ± 41	124 ± 17 **	74 ± 6.7	6.4 ± 1.8	180 ± 0.1
Milk	271 ± 18	371 ± 46	164 ± 3.5	52 ± 4.0	4.8 ± 1.6	67 ± 10
Bif-15	269 ± 23	314 ± 69	145 ± 17 *	85 ± 2.6 *	6.2 ± 2.7	250 ± 47 *
Bif-30	414 ± 47 *	469 ± 85	225 ± 5.6	70 ± 5.8	7.4 ± 0.7	128 ± 28
Bif-39	329 ± 49	257 ± 33	158 ± 19	63 ± 3.4	5.9 ± 0.4	102 ± 31

Each value represents the mean ± SE (*n* = 4). * *p* < 0.05 and ** *p* < 0.01, according to the Dunnett’s test, compared with the water group. TG, triglyceride; TC, total cholesterol; HDL-C, high-density lipoprotein-cholesterol.

**Table 2:** Composition of organic acids and viable cell count in Bifidobacterium-fermented milk.

	Culture (hr)	Concentrations of Organic Acids (%)						(cfu/mL)
		Citric Acid	Malic Acid	Succinic Acid	Lactic Acid	Formic Acid	Acetic Acid	
Milk	**-**	0.219	0.004	0.002	0.002	0.001	0.002	-
Control *B. longum*	24	0.211	0.007	0.019	0.525	0.004	0.562	-
Bif-15	48	0.191	0.006	0.008	0.482	0.004	0.555	1.2 × 10^7^
Bif-30	22	0.207	0.005	0.007	0.553	0.002	0.623	7.6 × 10^6^
Bif-39	21	0.213	0.004	0.007	0.541	0.003	0.602	1.4 × 10^9^

**Table 3:** List of PCR primer sequences.

Gene	Forward	Reverse
β-actin (*actb*)	GGAGATTACTGCCCTGGCTCCTA	GACTCATCGTACTCCTGCTTGCTG
ChREBP (*Mlxipl*)	GAAGACCCAAAGACCAAGATGC	TCTGACAACAAAGCAGGAGGTG
SREBP (*Srebp-1c*)	AGCACAGCAACCAGAAACTC	AGGTTTCATGCCCTCCATAG
L-type pyruvate kinase (*LPK*)	AACCTCCCCACTCAGCTACA	CCCTTCACAATTTCCACCTC
Acetyl-CoA carboxylase (*ACC*)	TACAACGCAGGCATCAGAAG	TGTGCTGCAGGAAGATTGAC
Fatty acid synthase (*Fas*)	CAGGAACAACTCATCCGTTCTCT	GGACCGAGTAATGCCGTTCA
GPR43 (*ffar2*, *GPR43*)	CAGAGGAGAACCAGGTGGAAG	GGCAGGGACCCCAGTAAGAA
PGC-1α (*ppargc1a*)	GACCCCAGAGTCACCAAATGA	GGCCTGCAGTTCCAGAGAGT
MEF2A (*mef2a*)	ATGAGAGGAACCGACAGGTG	TATCCGAGTTCGTCCTGCTT
Succinate dehydrogenase (*sdha*)	TGGGGCGACTCGTGGCTTTC	CCCCGCCTGCACCTACAACC
NADH dehydrogenase 1, mitochondrial (*mt-Nd1*)	CTCCCTATTCGGAGCCCTAC	ATTTGTTTCTGCTAGGGTTG

## Data Availability

Data are contained within the article.
